# Transmission dynamics of cholera in Yemen, 2017: a real time forecasting

**DOI:** 10.1186/s12976-017-0061-x

**Published:** 2017-07-26

**Authors:** Hiroshi Nishiura, Shinya Tsuzuki, Baoyin Yuan, Takayuki Yamaguchi, Yusuke Asai

**Affiliations:** 1 Graduate School of Medicine, Hokkaido University, Kita 15 Jo Nishi 7 Chome, Kita-ku, Sapporo, 060-8638 Japan; 2 CREST, Japan Science and Technology Agency, 4-1-8, Honcho, Kawaguchi-shi, Saitama, 332-0012 Japan

**Keywords:** *Vibrio cholerae*, Outbreak, Epidemiology, Prediction, Backcalculation

## Abstract

**Background:**

A large epidemic of cholera, caused by *Vibrio cholerae*, serotype Ogawa, has been ongoing in Yemen, 2017. To improve the situation awareness, the present study aimed to forecast the cholera epidemic, explicitly addressing the reporting delay and ascertainment bias.

**Methods:**

Using weekly incidence of suspected cases, updated as a revised epidemic curve every week, the reporting delay was explicitly incorporated into the estimation model. Using the weekly case fatality risk as calculated by the World Health Organization, ascertainment bias was adjusted, enabling us to parameterize the family of logistic curves (i.e., logistic and generalized logistic models) for describing the unbiased incidence in 2017.

**Results:**

The cumulative incidence at the end of the epidemic, was estimated at 790,778 (95% CI: 700,495, 914,442) cases and 767,029 (95% CI: 690,877, 871,671) cases, respectively, by using logistic and generalized logistic models. It was also estimated that we have just passed through the epidemic peak by week 26, 2017. From week 27 onwards, the weekly incidence was predicted to decrease.

**Conclusions:**

Cholera epidemic in Yemen, 2017 was predicted to soon start to decrease. If the weekly incidence is reported in the up-to-the-minute manner and updated in later weeks, not a single data point but the entire epidemic curve must be precisely updated.

## Background

Cholera is a bacterial disease caused by infection of small intestine by *Vibrio cholerae*, characterized by a variety of diarrhea, abdominal cramp and dehydration. Most common route of infection is through contaminated water and foods, and due to the environmental nature of transmission, the control is complicated in tropical environment where clean water is not easily accessible. From 2016, a large epidemic of cholera has been seen in Yemen, and stool samples have tested positive for *V. cholerae*, serotype Ogawa. The incidence once declined in early 2017, but a bigger epidemic was triggered from early-mid April 2017 with unprecedented size of cases [1]. Essential resources including vaccines, fluids and antibiotics have been allocated.

Mathematical modeling studies have contributed to better understanding of the cholera transmission dynamics [2–7]. Many models during the 2010 Haitian cholera outbreak explicitly accounted for the presence of asymptomatic individuals and also the transmission through water environment, because these features, especially the former have been the issue of unrecognized part of the outbreak [8]. Models have been frequently utilized for theoretically optimizing resource allocation of antibiotics and oral vaccines [2–8] in collaboration with public health experts [9].

Such modeling effort has been extended to real-time analysis and explicit assessment of predictive ability during the outbreak in Haiti [10, 11]. However, in the context of Yemen epidemic 2017, a modeling effort for both now-casting and forecasting, elevating situation awareness of both experts and the public, has yet to be made using a parsimonious modeling approach, possibly with real time updates. The present study aims to forecast the cholera epidemic in Yemen, 2017, explicitly addressing the reporting delay and ascertainment bias.

## Methods

### Epidemiological data

The present study analyzed the 2017 epidemic that we assumed to have started from 16 April 2017 (the first date of week 16) which coincided with a new increase in the reported incidence in 2017. Situation report of the cholera outbreak, as represented by Weekly Update and Epidemiology Bulletin, has been managed by the World Health Organization (WHO) Regional Office for the Eastern Mediterranean Regional Office, and the present study used weekly counts of suspected cases and deaths as reported in the Epidemiology Bulletin [12]. Suspected case of cholera adhered to the WHO’s classical case definition. That is, a case of cholera was suspected when a patient aged 5 years or more develops acute watery diarrhea, with or without vomiting [13]. The so-called case fatality risk (CFR), which was calculated in each week as the ratio of weekly count of deaths to that of cases, was also retrieved.

### Modeling methods

Let *c*_t,∆t_ be weekly “reported” incidence in week *t* which took place in ∆*t* days since the first date of corresponding week: we account for ∆*t* because ∆*t* greatly varied by Epidemiology Bulletin. Let *j*_t_ be the actual (unbiased) incidence in week *t*. That is, here we explicitly distinguish reported incidence *c* from actual incidence *j*. Setting the latest value of CFR in week 26 as the baseline (i.e. 1.0), the relative CFR of week *t* is denoted by *α*_t_. *F*_∆t_ is the cumulative distribution function of the time from illness onset to reporting. We assume that the actual (unbiased) CFR is a constant without any interventions or treatment and that all deaths are reported. Moreover, we assume that *α*_t_ in and after week 26 remains to be the value of 1. The expected value of the weekly incidence is described as

1 $$ E\left[{c}_{t,\varDelta t}\right]=\frac{j_t}{\alpha_t}{F}_{7\left(s-t\right)+\varDelta t}, $$

where *s* represents the latest week of observation (for *t* ≤ *s*). *F*_∆t_ was assumed to follow an exponential distribution with mean *δ* days, i.e., *F*_∆*t*_ = 1-exp(−∆*t* /*δ*), and thus, the product of *j* and *F* captures the reporting delay structure in empirical data. *F*_∆t_ is important in interpreting the updated epidemic curve every week. As we assume that the WHO’s weekly CFR estimate mirrors the frequency of case ascertainment in each week, *α*_t_ adjusts time-dependent variations in case ascertainment. The unbiased incidence, *j*_t_ in week *t* is obtained from the cumulative incidence, *J*(*t*):

2 $$ {j}_t=J(t)-J\left(t-1\right), $$

where *J*(*t*) was assumed to be described by two different parsimonious models. One is the logistic growth curve, i.e.,

3 $$ J(t)=\frac{K}{1+ \exp \left(-r\left(t-{t}_i\right)\right)}, $$

where *K* is referred to as the carrying capacity, representing the cumulative incidence at time infinity, i.e., our interest in the present study, *r* is the growth rate and *t*_i_ is the point of inflection, corresponding to the peak timing of an epidemic. The other model is a slightly more general model, referred to as the generalized logistic model or the so-called “Richards model”, i.e.,

4 $$ J(t)=\frac{K}{{\left[1+g \exp \left(-r\left(t-{t}_i\right)\right)\right]}^{\frac{1}{g}}}, $$

where *g* is the parameter that partially determines the point of inflection on vertical axis. The model (4) is known as flexible with only 4 parameters and has been widely applied to capture the temporal distribution of epidemics of variety of diseases [14–17].

Assuming that the observed data in week *t*, *c*_t,∆t_, follows a Poisson distribution, the likelihood function to estimate parameters of abovementioned models (i.e., 1 parameter for *F* and 3 or 4 parameters for *J*) is

5 $$ L\left(\theta; D\right)=\frac{E{\left[{c}_{t,\varDelta t}\right]}^{c_{t,\varDelta t}} \exp \left(-E\left[{c}_{t,\varDelta t}\right]\right)}{c_{t,\varDelta t}!}, $$

where *θ* represents the population parameter and *D* the observed data.

The maximum likelihood estimates were obtained by minimizing the negative logarithm of (5). Profile likelihood based 95% confidence intervals (CI) were used. To compare model (3) and (4), Akaike Information Criterion (AIC) was employed.

Given empirical data for each observed week, we implemented real time forecasting and sequentially updated it every week. The latest forecast that we present is based on the dataset from week 16 to 26, 2017. The forecast of unbiased cumulative number of cases was obtained from parameterized models (3) and (4). Additionally adjusting the reporting delay, we also produced forecasts of weekly incidence that is expected to be observed in week 27, 28, 29 and 30 in WHO’s report.

### Ethical considerations

The present study analyzed data that is publicly available. As such, the datasets used in our study were de-identified and fully anonymized in advance, and the analysis of publicly available data without identity information does not require ethical approval.

## Results

**Fig. 1:**
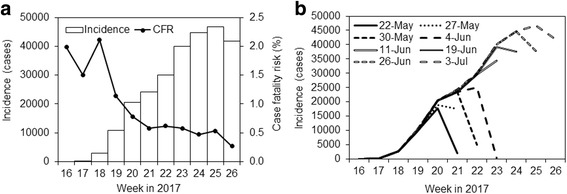
Weekly incidence of the cholera epidemic, Yemen, 2017. **a** Weekly number of suspected cholera cases (*left vertical axis*) along with the case fatality risk (CFR) calculated as the ratio of weekly count of deaths to cases. Week 16 on *horizontal axis* corresponds to 16 April 2017. **b** Reported and updated epidemic curves from 22 May to 3 July 2017. Each epidemic curve represents the weekly number of suspected cases reported by the date of report (i.e., 22, 27, 30 May, 4, 11, 19 and 26 June and 3 July, respectively). Differences in the number of cases reported in the same week between two or more curves reflect the delay in reporting. Week 16 on horizontal axis corresponds to 16 April 2017

Figure 1 shows the epidemic curve. The incidence continuously increased from week 16, the first date of which is 16 April 2017 which we use as the initial date of the 2017 epidemic in the following analysis. Counting from 27 April 2017 (from which WHO counted [12]), a total of 356,591 suspected cases and 1802 deaths were reported as of 17 July 2017. The ratio of weekly deaths to cases (i.e., the so-called CFR in the WHO’s report) in the week of 25 June 2017 was 0.27, while that in the week of 16 April 2017 was 1.98. Setting the latest value of CFR (=0.27) as the baseline, those in the week of 16th, 23rd and 30th of April 2017, *α*_t_, were 7.39, 5.62 and 7.85, respectively, which we assume that they reflect small case ascertainment in April. Incidence of the latest week 26 in Fig. 1a may look indicating that the epidemic turned into decreasing trend, but Fig. 1b implicates that always the incidence in the latest week is underestimated in its initial report due to reporting delay. In the following week, the current weekly incidence likely increases.

**Fig. 2:**
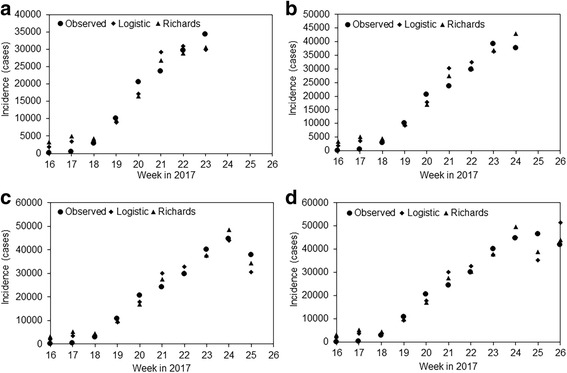
Real time forecasts of suspected patients of cholera epidemic, Yemen, 2017. The weekly reported incidence that were released on 11, 19, 26 June and 3 July 2017 were compared with forecasts of logistic model and Richards model (i.e., generalized logistic model) on figures **a**), **b**), **c**) and **d**), respectively. Week 16 in 2017 corresponds to the week starting from 16 April and it is the beginning of the second wave of cholera epidemic in Yemen, 2017

Figure 2 shows the real-time fitting results of our model. Both logistic and generalized logistic curves qualitatively captured the observed patterns of reported incidence, and that the overall patterns of agreement have not varied with time. AIC values of using the latest data were 1,986,063 and 2,045,441 for logistic and generalized logistic models, respectively, favoring the simpler model (3). The estimated mean reporting delay were 4.4 days (95% CI: 3.2, 5.8) and 4.0 (95% CI: 2.9, 5.3) days for logistic and generalized logistic models, respectively, and the growth rate was 0.06 (95% CI: 0.05, 0.07) per day and 0.18 (95% CI: 0.09, 0.65) per day, respectively, for these models. Parameter *g* of Richards model was estimated at 6.9 (95% CI: 2.4, 33.2).

**Fig. 3:**
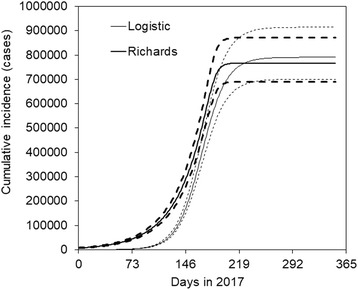
Predicted actual cumulative incidence of cholera epidemic, Yemen, 2017. Days (*horizontal axis*) are counted from 1 January 2017. For both forecasts (one using logistic model and the other using Richards (or generalized logistic) model), the 95% confidence intervals, *dashed lines*, were obtained using the 95% confidence intervals of carrying capacity (note that this is not the result from Bootstrapping of all parameters for simplicity)

Figure 3 shows the forecasted *J*(*t*), representing the actual cumulative incidence. Carrying capacity, or the cumulative incidence at the end of the epidemic, was estimated at 790,778 (95% CI: 700,495, 914,442) cases and 767,029 (95% CI: 690,877, 871,671) cases, respectively, by using logistic and generalized logistic models. Counting days from 1 January 2017 as day 0, the inflection point was estimated at day 168.0 (95% CI: 165.6, 171.1) and 173.3 (95% CI: 169.1, 180.7), respectively, for these models, indicating that the latest week (i.e. week 26) is about the time just after experiencing the inflection point of the epidemic curve.

**Fig. 4:**
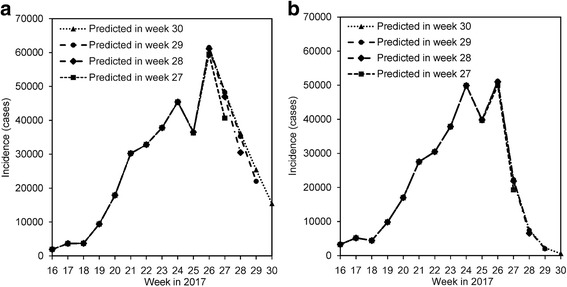
Predicted weekly reported incidence to be reported in week 27, 28, 29 and 30, 2017. **a** Logistic model and **b** Richards (generalized logistic) model. Parameter estimates of both models were obtained from the datasets from week 16 to 26

Figure 4 shows the forecasting result of observed incidence data using model (1), as derived from logistic (Fig. 4a) and generalized logistic (Fig. 4b) models. The weekly incidence that is expected to be reported in week 27 and afterwards are predicted to decrease over time. The logistic curve yielded slower decline with evident reporting delay compared with Richards model.

## Discussion

The present research study responded to the cholera epidemic in Yemen, 2017 in real time. Using the weekly incidence of suspected cases, updated as a revised epidemic curve every week, the reporting delay was explicitly incorporated into the model. Moreover, using the CFR (as calculated by the WHO which was implemented in a biased manner [18]), ascertainment bias was adjusted, enabling us to parameterize the family of logistic curves for modeling the unbiased incidence. As a result, it was estimated that we have just passed through the epidemic peak by week 26, and the unbiased cumulative incidence was forecasted to range from 690 to 910 thousand cases by the end of the epidemic. Doing so, while high incidence nearby epidemic peak may lead Yemen’s people to feel that the epidemic is somewhat uncontrollable due to massive number of reported cases, we have been able to objectively demonstrate that the epidemic peak is likely over and elevate people’s situation awareness through our “now-casting” approach.

To our knowledge, this modeling study is the first to elevate the situation awareness of the cholera epidemic in Yemen, 2017 via statistical forecasting of the epidemic. Employing parsimonious models with small number of parameters, the observed patterns of incidence and reporting were appropriately captured and we were successful in obtaining real time forecasts. While the epidemic peak was not directly identifiable as of week 26, 2017 only from reported data, the model has radically indicated that the inflection point has plausibly been passed. Moreover, jointly using the relative CFR with the abovementioned model with reporting delay, we assumed that the time period with high CFR involved relatively low ascertainment, enabling us to strongly feel that the qualitative patterns of unbiased epidemic curve can be captured.

Epidemiological modelers will experience similar epidemics in the future. What to be learnt from our modeling exercise are two folds. First, if the weekly incidence is reported in the up-to-the-minute manner and updated in later weeks, not a single data point but the entire epidemic curve must be precisely updated-and-reported. In the Yemen case, we initially intended to implement real time modeling using daily incidence data, but this intent was in vain mainly due to the absence of regular updates in the epidemic curves showing daily incidence. Second, not only the epidemic curve, but death data should also be consistently updated over time, so that it helps researchers to adjust ascertainment bias.

Carrying capacity, or the cumulative incidence at the end of the epidemic, is worthy of further discussions. We estimated that *K* ranges from 690 to 910 thousand cases, but that value is dependent on our choice of the weekly CFR in week 26 (i.e. CFR = 0.27). If the actual CFR is greater than 0.27, our estimated *K* may be an overestimate. On the other hand, considering that the empirically observed CFR does not capture mild and asymptomatic infected individuals that are never reflected in the denominator of the CFR estimation, the estimated *K* may be a considerable underestimate.

Several limitations must be noted. First, we were not able to assure the quality of suspected cases at an individual level. Sometimes, weekly incidence in a specific week has decreased (rather than increase due to reporting delay) as the epidemic curve was updated, and in such an instance, we had to adopt the smallest latest value regardless of the week of update, because we had to avoid any decrease in the observed incidence as a function of week through minimum arbitrariness for adjustment. Second, the validity of forecast is limited due to the use of parsimonious phenomenological model. Prediction approaches using more mechanistic models would be beneficial. Third, our analysis rests on the dataset of entire Yemen, and we have not been able to dig into more detailed heterogeneous data. Spatio-temporal heterogeneity is known to play a key role in cholera transmission [19, 20] and that could allow examining the impact of environmental predictors on the transmission dynamics [21]. Fourth, our model cannot incorporate the population impact of interventions on the transmission dynamics, due to phenomenological nature of the model that we used.

While several modeling research subjects remain, the present study acted as the first to contribute to improving situation awareness of cholera epidemic in Yemen, 2017. Although the epidemic is predicted to start to decline, it is vital that ongoing resource allocations and countermeasures are tightly conducted to minimize potential number of victims.

## Conclusions

The present study responded to the cholera epidemic in Yemen, 2017 in real time. Employing parsimonious models with small number of parameters, the observed patterns of incidence and reporting were appropriately captured. It was estimated that we have just passed through the epidemic peak by the end of week 26, and the weekly incidence was predicted to start to decrease in the following weeks. The unbiased cumulative incidence was forecasted to range from 690 to 910 thousand cases by the end of the epidemic.

## Abbreviations

AIC: Akaike Information Criterion
CFR: Case fatality risk
CI: Confidence interval
WHO: World Health Organization
